# Complete Genome Sequence of Mycobacteriophage Faze9

**DOI:** 10.1128/MRA.01372-19

**Published:** 2020-01-16

**Authors:** John Grossi, John Shebby, Christopher W. Brey

**Affiliations:** a Department of Science, Math, and Computer Science, Marywood University, Scranton, Pennsylvania, USA; DOE Joint Genome Institute

## Abstract

Mycobacteriophage Faze9 is a novel lytic phage that was isolated from soil collected in Scranton, PA, using the host Mycobacterium smegmatis mc^2^155. Faze9 has a double-stranded DNA genome composed of 67,503 kbp, has a G+C content of 68.9%, encodes 91 predicted proteins, and is closely related to mycobacteriophages in the B2 subcluster.

## ANNOUNCEMENT

Bacteriophage Faze9 was isolated from the host organism Mycobacterium smegmatis mc^2^155. M. smegmatis mc^2^155 is a Gram-positive, acid-fast, rod-shaped aerobic bacterium. M. smegmatis is a nonpathogenic bacterium that serves as the model system for the analysis of Mycobacterium tuberculosis genes (1). Faze9 was isolated from soil collected in Scranton, PA (41.4319N, 75.6322W), using an enrichment protocol (2) and the bacterial host M. smegmatis mc^2^155, by the Marywood University phage hunters as part of the Science Education Alliance-Phage Hunters Advancing Genomics and Evolutionary Science (SEA-PHAGES) program of the Howard Hughes Medical Institute (3). Plaques were clear and approximately 1 mm in diameter.

Faze9 is a member of actinobacteriophage subcluster B2. Based on morphological analysis using electron microscopy, Faze9 exhibits a *Siphoviridae* morphotype with an icosahedral head diameter of approximately 48 nm and a tail length of 283 nm. Genomic DNA was isolated from Faze9 lysate with a Wizard DNA extraction kit (Promega), following the kit protocol. A sequencing library was prepared from genomic DNA using a NEBNext Ultra II FS kit with dual-indexed barcoding and was run on an Illumina MiSeq system, which yielded ∼920,000 single-end 150-base reads, representing ∼1,962-fold coverage of the genome. The raw reads were assembled using Newbler 2.9, with default settings, and yielded a single phage contig, which was checked for completeness, accuracy, and phage genomic termini using Consed 29 and methods described previously (4). Faze9 contains a circularly permuted genome consisting of 67,503 kbp, with 68.9% G+C content and a predicted start codon distribution of 66% ATG and 34% GTG. The G+C contents of the host bacterium (M. smegmatis mc^2^155) and Faze9 were similar, being 67.4% and 68.9%, respectively (5). Genome annotation was performed manually after the completion of preliminary annotation by DNA Master 5.23.2 (http://cobamide2.bio.pitt.edu). Protein-coding gene prediction was executed by using Glimmer 3.02 (6) and GeneMark 2.5p (7), and functional assignments were made by employing NCBI BLASTP with the Conserved Domain Database at NCBI (8), Actinobacteriophage Database BLAST (https://phagesdb.org/blast), HHpred (https://toolkit.tuebingen.mpg.de/tools/hhpred) (9), and Phamerator (https://phamerator.org) (10). Annotations predicted 91 open reading frames with 24 known functional genes (24.6% of the genome); 5.1% of the Faze9 genome represented noncoding nucleotides. No tRNA genes were identified.

This genome contains genes with predicted functions related to phage “DNA” packaging (HNH endonuclease, head-to-tail adaptor, portal protein, and terminase), gene and virion structure (minor tail protein and tape measure protein), DNA replication (RuvC-like resolvase, DNA helicase, and DNA primase/polymerase), host lysis (lysin A and holin), and DNA modifications (ParB-like nuclease domain protein, queine tRNA-ribosyltransferase, QueC, QueD, and Que-like queosine (Genebank term) biosynthesis protein), as well as 67 hypothetical proteins. Faze9 shows genome-wide similarity to the other 11 lytic phages in this cluster, sharing 99.34% identity with its closest relative, Coffee (GenBank accession number MK620898). Comparison to Coffee was performed through a nucleotide BLAST search (https://blast.ncbi.nlm.nih.gov). The subcluster B2 bacteriophages Arbiter, Hedgerow, and LizLemon each had 99% similarity to Faze9, with 100% coverage (NCBI BLAST).

### Data availability.

The complete genome sequence of Faze9 was deposited in GenBank with accession number MK061411, BioProject accession number PRJNA488469, and SRA accession number SRX6867480.

## References

[1] Nuria A, Soto CY, Roca I, Martin C 2004 *Mycobacterium smegmatis* displays the *Mycobacterium tuberculosis* virulence-related neutral red character when expressing the Rv0577 gene. FEMS Microbiol Lett 231:283–289. 14987776 10.1016/S0378-1097(04)00008-4

[2] Poxleitner M, Pope W, Jacobs-Sera D, Sivanathan V, Hatfull G 2016 Phage discovery guide. Howard Hughes Medical Institute, Chevy Chase, MD seaphagesphagediscoveryguide.helpdocsonline.com/home.

[3] Jordan TC, Burnett SH, Carson S, Caruso SM, Clase K, DeJong RJ, Dennehy JJ, Denver DR, Dunbar D, Elgin SC, Findley AM, Gissendanner CR, Golebiewska UP, Guild N, Hartzog GA, Grillo WH, Hollowell GP, Hughes LE, Johnson A, King RA, Lewis LO, Li W, Rosenzweig F, Rubin MR, Saha MS, Sandoz J, Shaffer CD, Taylor B, Temple L, Vazquez E, Ware VC, Barker LP, Bradley KW, Jacobs-Sera D, Pope WH, Russell DA, Cresawn SG, Lopatto D, Bailey CP, Hatfull GF 2014 A broadly implementable research course in phage discovery and genomics for first year undergraduate students. mBio 5:e01051-13. doi:10.1128/mBio.01051-13. 24496795 PMC3950523

[4] Russell DA 2018 Sequencing, assembling, and finishing complete bacteriophage genomes, p 109–125. *In* Clokie MRJ, Kropinski AM, Lavigne R (ed), Bacteriophages: methods and protocols, vol 3 Springer, New York, NY. 10.1007/978-1-4939-7343-9_9 29134591

[5] Mohan A, Padiadpu J, Baloni P, Chandra N 2015 Complete genome sequences of *Mycobacterium smegmatis* laboratory strain (MC^2^ 155) and isoniazid-resistant (4XR1/R2) mutant strains. Genome Announc 3:e01520-14. doi:10.1128/genomeA.01520-14. 25657281 PMC4319614

[6] Delcher AL, Bratke KA, Powers EC, Salzberg SL 2007 Identifying bacterial genes and endosymbiont DNA with Glimmer. Bioinformatics 23:673–679. doi:10.1093/bioinformatics/btm009. 17237039 PMC2387122

[7] Besemer J, Borodovsky M 2005 GeneMark: Web software for gene finding in prokaryotes, eukaryotes and viruses. Nucleic Acids Res 33:W451–W454. doi:10.1093/nar/gki487. 15980510 PMC1160247

[8] Altschul SF, Gish W, Miller W, Myers EW, Lipman DJ 1990 Basic Local Alignment Search Tool. J Mol Biol 215:403–410. doi:10.1016/S0022-2836(05)80360-2. 2231712

[9] Söding J, Biegert A, Lupas AN 2005 The HHpred interactive server for protein homology detection and structure prediction. Nucleic Acids Res 33:W244–W248. doi:10.1093/nar/gki408. 15980461 PMC1160169

[10] Cresawn SG, Bogel M, Day N, Jacobs-Sera D, Hendrix RW, Hatfull GF 2011 Phamerator: a bioinformatic tool for comparative bacteriophage genomics. BMC Bioinformatics 12:395. doi:10.1186/1471-2105-12-395. 21991981 PMC3233612

